# Targeting Mdmx to treat breast cancers with wild-type p53

**DOI:** 10.1038/cddis.2015.173

**Published:** 2015-07-16

**Authors:** S Haupt, D Buckley, J-MB Pang, J Panimaya, P J Paul, C Gamell, E A Takano, Y Ying Lee, S Hiddingh, T-M Rogers, A F A S Teunisse, M J Herold, J-C Marine, S B Fox, A Jochemsen, Y Haupt

**Affiliations:** 1 Tumor Suppression Laboratory, Research Division, Peter MacCallum Cancer Centre, East Melbourne, Victoria, Australia; 2 Department of Pathology, Peter MacCallum Cancer Centre, East Melbourne, Victoria, Australia; 3 Department of Molecular Cell Biology, University Medical Centre, Leiden, The Netherlands; 4 Department of Molecular Genetics of Cancer, The Walter and Eliza Hall Institute, Parkville, Victoria, Australia; 5 Department of Medical Biology, University of Melbourne, Parkville, Victoria, Australia; 6 Center for Human Genetics, KU Leuven, Leuven, Belgium; 7 Sir Peter MacCallum Department of Oncology, University of Melbourne, Parkville, Victoria, Australia; 8 Department of Pathology, University of Melbourne, Parkville, Victoria, Australia; 9 Department of Biochemistry and Molecular Biology, Monash University, Clayton, Victoria, Australia

## Abstract

The function of the tumor suppressor p53 is universally compromised in cancers. It is the most frequently mutated gene in human cancers (reviewed). In cases where p53 is not mutated, alternative regulatory pathways inactivate its tumor suppressive functions. This is primarily achieved through elevation in the expression of the key inhibitors of p53: Mdm2 or Mdmx (also called Mdm4) (reviewed). In breast cancer (BrCa), the frequency of p53 mutations varies markedly between the different subtypes, with basal-like BrCas bearing a high frequency of p53 mutations, whereas luminal BrCas generally express wild-type (wt) p53. Here we show that Mdmx is unexpectedly highly expressed in normal breast epithelial cells and its expression is further elevated in most luminal BrCas, whereas p53 expression is generally low, consistent with wt p53 status. Inducible knockdown (KD) of Mdmx in luminal BrCa MCF-7 cells impedes the growth of these cells in culture, in a p53-dependent manner. Importantly, KD of Mdmx in orthotopic xenograft transplants resulted in growth inhibition associated with prolonged survival, both in a preventative model and also in a treatment model. Growth impediment in response to Mdmx KD was associated with cellular senescence. The growth inhibitory capacity of Mdmx KD was recapitulated in an additional luminal BrCa cell line MPE600, which expresses wt p53. Further, the growth inhibitory capacity of Mdmx KD was also demonstrated in the wt p53 basal-like cell line SKBR7 line. These results identify Mdmx growth dependency in wt p53 expressing BrCas, across a range of subtypes. Based on our findings, we propose that Mdmx targeting is an attractive strategy for treating BrCas harboring wt p53.

The p53 tumor suppressor protein is a key factor in the cellular stress response.^1, 2^ Functional p53 prevents the progression of cancer by mounting growth inhibition in the form of apoptosis, senescence and/or autophagy.^3^ The exact tumor suppressive functions of p53 that prevent cancer are currently the subject of extensive studies (reviewed in Bieging *et al.*^4^). These growth inhibitory functions are frequently lost during tumorigenesis, most commonly through direct mutations in the *p53* gene, which occur in ~50% of all human cancer cases.^5^ However, in the remaining cases, p53 status remains wild type (wt) and its function and/or expression is compromised by other mechanisms. The two major nonredundant inhibitors of p53 are the Mdm proteins: Mdm2 and Mdmx (also called Mdm4).^6, 7^

Mdm2 is the major E3 ligase of p53, promoting its ubiquitination and proteasomal degradation.^8, 9^ Mdmx in contrast, inhibits the transcriptional activity of p53 and enhances the ability of Mdm2 to target p53 for degradation, although it does not have an E3 ligase activity of its own.^10^ Both Mdm2 and Mdmx expression are elevated in various cancer types. For example, Mdm2 is amplified in the majority (70%) of well-differentiated liposarcomas,^11^ whereas the Mdmx protein is elevated in most melanomas and retinoblastoma (~70%).^12, 13^ In these cases, elevation of these Mdm proteins directly correlates with wt p53 status. In contrast, Yu *et al.*^14^ reported elevated protein levels of Mdm2 (38%) and Mdmx (65%), and low-level *Mdmx* gene amplification (56.5% identified by fluorescence *in situ* hybridization (FISH)) in an apparently wt p53 context (as suggested by an absence of allelic loss and no increased protein detection) in primary breast cancers (BrCas). This contrasted with the far more modest levels (5%) previously described.^15^ The discrepancy between these findings is apparently due to what is considered amplification, where the former included low-level amplifications.^14^

An overall p53 mutation rate approaching 30% defines it as the most common genetic alteration in BrCa. However, the mutational frequency is highly dependent on the cancer subtype. Specifically, p53 mutations have been reported in 88% of basal-like BrCas, ~70% of apocrine carcinomas and in ~50% of HER2-amplified tumors. In the more common luminal subtypes, p53 mutations are reported in ~15% of luminal A and ~40% of luminal B subtype. Moreover, the nature of p53 mutations also differ between subtypes, with basal-like BrCa and apocrine cancers having complex p53 mutations characterized by ‘insertion/deletion polymorphisms', whereas luminal tumors are generally simpler base substitutions.^16, 17, 18^

In this study we tested whether elevated Mdmx expression can account for the tolerance of wt p53 in BrCas, and whether downregulation of Mdmx is an efficient approach to targeting BrCas bearing wt p53. We found that Mdmx is highly expressed in BrCas. We showed that ablation of Mdmx impeded the growth of luminal BrCa cell line MCF-7 in culture, in a p53-dependent manner. Growth inhibition in response to Mdmx knockdown (KD) was replicated in an additional wt p53 expressing luminal cell line MPE600. In an orthotopic model of human luminal BrCa using MCF-7 cells, Mdmx KD was demonstrated to both prevent tumor initiation and also to inhibit progression in established tumors. Importantly, our findings extend beyond luminal BrCas and identified that in the wt p53 basal-like cell line SKBR7, Mdmx KD is also growth inhibitory. Our study strongly supports the notion that targeting Mdmx has a therapeutic potential across a range of BrCa subtypes expressing wt p53.

## Results

### Mdmx levels are elevated in luminal BrCa corresponding with low p53 levels

**Figure 1:**
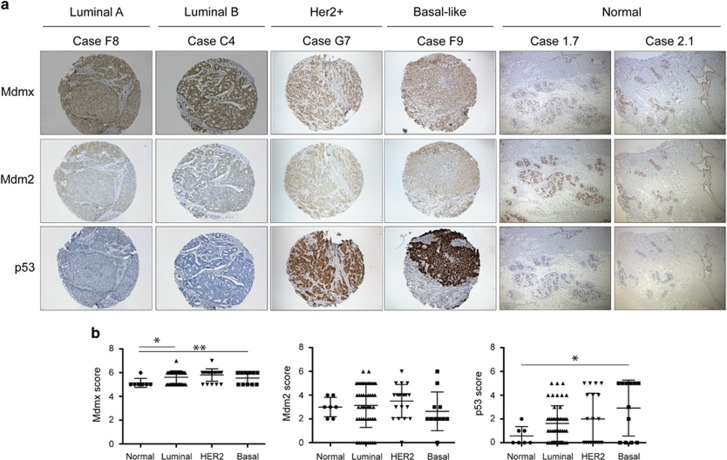
Elevated Mdmx expression inversely correlated with p53 levels. TMA from luminal, Her 2-positive (Her2+), basal-like BrCas and normal breast were stained for Mdmx (top row), Mdm2 (middle row) and p53 (lower row). Representative samples from the 81 tumor biopsies (for luminal A (Case F8) and luminal B (Case C4), Her2+ and Basal subtypes) and 6 normal breast tissues are shown in **a**. Tissues were scored according to the proportion of cells stained and the overall intensity in the nucleus, and the findings are presented in **b** according to BrCa subtypes. Staining intensity ranged from minimum to maximum of 0 to 3, whereas the proportion of cells stained was according to an elevating scale of 0 to 4 (0 to >80%, respectively, as described in the text) and scores were summed to a final possible maximum score of 8. Statistical significance discriminated the Mdmx normal and luminal samples (S.D., *P*=0.0258) and also normal and Her2 samples (*P*=0.0055). p53 staining only differed significantly from normal levels in the basal samples (*P*=0.0216). All the experiments were repeated at least three times. Errors bars represent S.D. of the mean. *P*-values were calculated by Student's *t*-test

Wt p53 status in cancers is often associated with elevated Mdm proteins (reviewed in Wade *et al.*^6^). To explore whether gene copy number of Mdm2 and/or Mdmx are abnormal in BrCas, we measured gene copy amplification by FISH in a BrCa tissue micro-array (TMA). The TMA comprised 81 BrCa biopsies: luminal (*n*=50; 62%), Her2 (*n*=20; 25%), basal (*n*=11; 13%) and a panel of normal breast tissues. As shown in Supplementary Figure 1A, none of these BrCa samples had Mdm2 probe amplification and only a single luminal sample had Mdmx probe amplification (Supplementary Figure 1B). Immunohistochemistry (IHC) staining of Mdm2, Mdmx and p53 protein levels were compared in BrCa TMA samples relative to normal controls. Unexpectedly, we observed high Mdmx and Mdm2 expression levels in the normal breast ductal epithelial cells relative to neighboring cells (Figure 1, see Discussion). Mdm2 staining was detected in both the nucleus and the cytoplasm of many of the BrCa samples (representative luminal, Her2+ and basal-like samples in Figure 1a) and high levels were quantified in the nucleus (Figure 1b). Mdm2 protein in the luminal tumors was present at similar levels to the normal controls, consistent with no amplification of the *mdm2* gene. The majority of the luminal samples exhibited low levels of p53 protein, suggestive of wt p53 status and were consequently chosen as the main focus of our study. Significantly higher levels of Mdmx protein were identified in the luminal tumor samples compared with the normal samples (Student's *t*-test *P*=0.0258; Figure 1b). Strong nuclear Mdmx expression was detected in the majority of the cancer cells in the tumor biopsies, but not in the adjacent normal tissue, or the stroma (Figure 1a). Most luminal samples with high Mdmx expression had low p53 expression, suggestive of wt p53 status. Luminal samples were further stratified according to Ki67 staining^19^ into luminal A (<14% Ki67 positive) and B subtype (≥14% Ki67 positive), and Mdmx was high across both (i.e., Mdmx staining did not discriminate luminal A and B subtypes; as evident in Figure 1a). Taken together, these analyses of luminal BrCa biopsies show a strong correlation between low p53 expression levels and elevated levels of Mdm proteins. These findings are consistent with previous studies demonstrating elevated expression of the Mdm proteins in BrCa cell lines with wt p53 status.^20^

### Downregulation of Mdmx in luminal BrCa MCF-7 cells impedes growth *in vitro* in a p53-dependent manner

**Figure 2:**
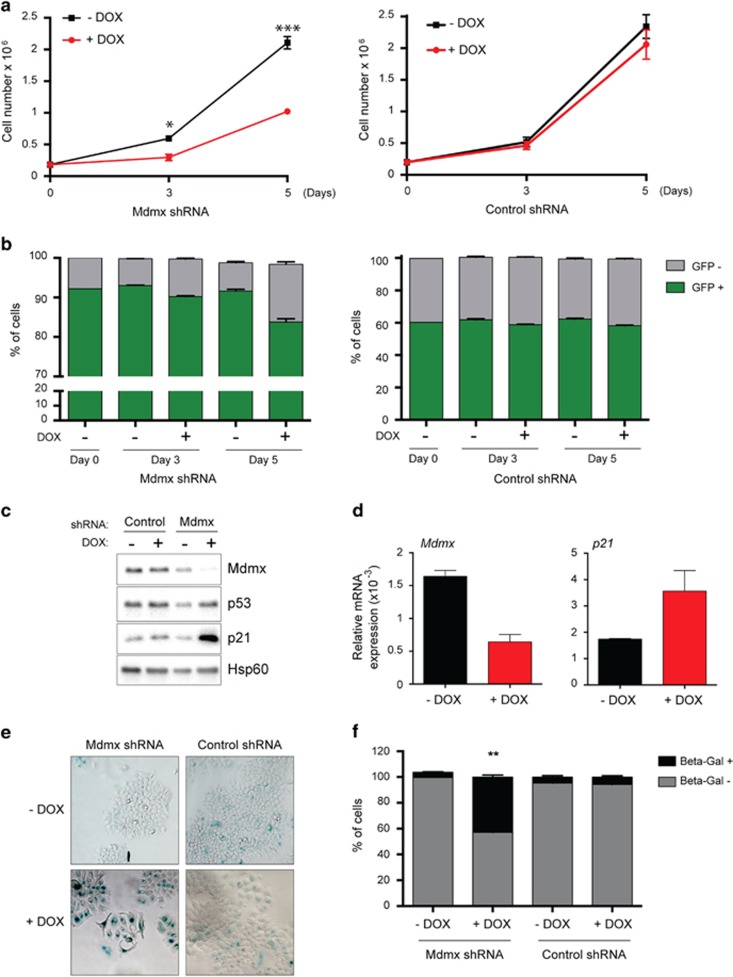
KD of Mdmx in MCF-7 cells attenuated cell proliferation by activating p53. MCF-7 cells expressing Mdmx shRNA or control shRNA (shMdmx wobble control) were treated with DOX (80 ng/ml) and cell numbers were quantified at 3 and 5 days using volumetric counting on the FACs VERSE flow cytometer. Statistical significance was only measured after DOX treatment of Mdmx shRNA samples (3 days: *P*=0.0041; 5 days *P*=0.0004) (**a**). The proportion of cells expressing the shRNA constructs were determined by GFP positivity at different days following treatment using flow cytometry (**b**). Protein expression of Mdmx, p53 and p21, PML and the loading control HSP60 were determined by western blotting at day 3 of treatment (**c**). RNA expression of Mdmx and p21 were determined at day 3 of treatment by RT-PCR. Each experiment in **a**, **b** and **d** represents triplicate data. All the experiments were repeated at least three times. Errors bars represent S.D. of the mean. *P*-values were calculated by Student's *t*-test. Senescence after 5 days of DOX treatment led to significant senescence only in shMdmx cells and is indicated by *β*-gal staining (**e**), and quantified as significant by Student's *t*-test *P*-value<0.0001 (**f**)

To examine whether Mdmx expression promotes the growth of luminal wt p53 BrCa cells, we chose MCF-7 as the primary model system, as it has wt p53 and relatively high Mdmx levels. The expression of *Mdmx* was knocked down by lentiviral-mediated doxycycline (DOX)-inducible shRNA expression. Controls included an empty vector and a wobble shRNA (identical sequence but with two substitutions) to Mdmx. Mdmx KD was induced with DOX, and in MCF-7 cells reduced cell numbers were evident after 3 days and further exacerbated after 5 days (>50% Figure 2a). As this lentiviral vector also expresses GFP, we used fluorescence to discriminate how transduced cells were effected by Mdmx KD (where nontransduced cells in the population were GFP negative). This analysis revealed that Mdmx KD (after 5 days) reduced viability of GFP-positive cells by <10% (indicating that cell death was not the major cause of the reduced cell numbers (Figure 2a)), whereas the control shRNA had no significant effect (Figure 2b). DOX treatment of MCF-7 parental cells or cells infected with the lentiviral vector control also had no effect on cell growth (Supplementary Figure 2A and Figure 2b, respectively). The growth inhibitory effects of Mdmx KD were reiterated using additional Mdmx shRNA sequences: shRNA 1 with 3 and 5 days of DOX induction (Supplementary Figures 3A–D, western blot, densitometric quantification, reduction in live cell numbers, respectively) and shRNA II with 3 and 6 days of DOX induction (Supplementary Figures 3E–G, western blot, densitometric quantification, reduction in live cell numbers, respectively, without significant increase in death).

To measure the effect of DOX treatment on Mdmx levels, cells were treated for 3 days with DOX, or controls were left untreated, before Mdmx protein staining by western blotting. Mdmx levels were significantly reduced in the presence of DOX (Figure 2c), which correlated with elevated expression of the p53 target gene *p21*, which is growth inhibitory. p53 levels remained relatively unchanged compared with the shRNA control (Figure 2c). Reduction in *Mdmx* messenger RNA (mRNA) levels and concurrent induction of *p21* mRNA levels were confirmed by quantitative PCR (QPCR) (Figure 2d). Senescence growth inhibition was suggested by the elevation of levels of p21 protein in response to shMdmx induction, together with *β*-galactosidase (*β*-gal) staining of cells (Figures 2e and f).

**Figure 3:**
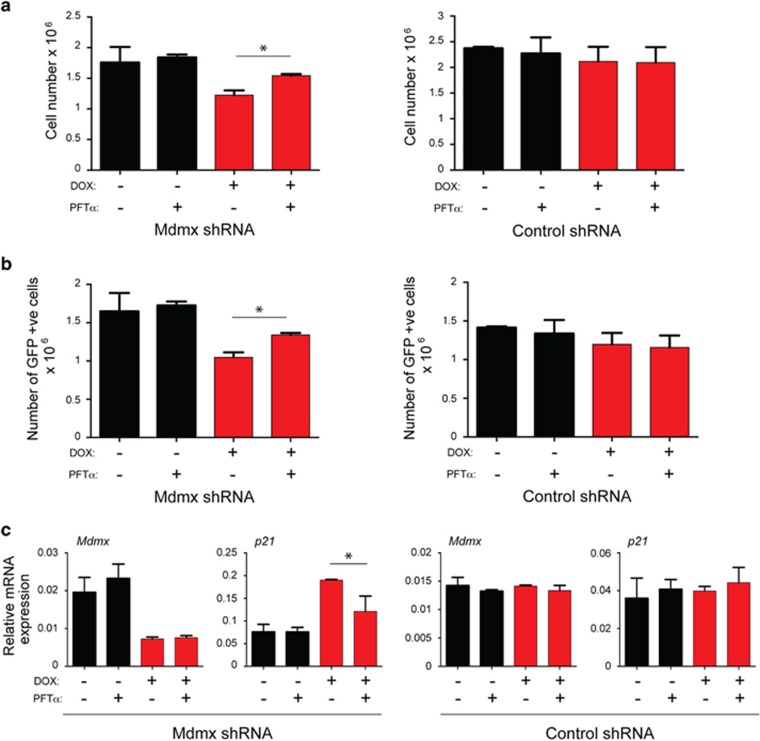
KD of Mdmx impeded MCF-7 cells in a p53-dependent manner. MCF-7 cells expressing Mdmx shRNA or control shRNA (shMdmx wobble control) were treated and analyzed as described in Figure 2, with the exception that half the samples were also treated with the p53 inhibitor pifithrin-*α* (2.5 *μ*M PFT*α*). For the analysis of cell numbers (**a**) and GFP positivity (**b**), cells were treated for 5 days. PFT*α* offered statistically significant protection against Mdmx shRNA, both for cell numbers (**a**, *P*=0.0027) and also for numbers of GFP positive cells (**b**, *P*=0.0018). For the measurement of Mdmx and p21 expression, cells were treated for 3 days and analyzed by RT-PCR (**c**). All the experiments represent triplicates. Errors bars represent S.D. of mean. *P*-values were calculated by Student's *t*-test

To demonstrate more directly the role of p53 in the effect of Mdmx KD, the p53 inhibitor pifithrin-*α* (PFT*α*) was included. PFT*α* partially relieved the effect of Mdmx on total cell viability (Figure 3a), as evident from the proportion of viable GFP-positive cells (Figure 3b) and the unaltered expression of the p53 target gene *p21* (Figure 3c). Of note, treatment of cells with PFT*α* did not interfere with the *Mdmx* KD nor did it affect p53 levels (data not shown). Together, these experiments demonstrate that treatment of cells with PFT*α* partially rescued the cells from the effect of Mdmx KD, which supports the role of p53 in the observed effects of Mdmx KD.

### Downregulation of Mdmx impedes growth of BrCa cells in mice

**Figure 4:**
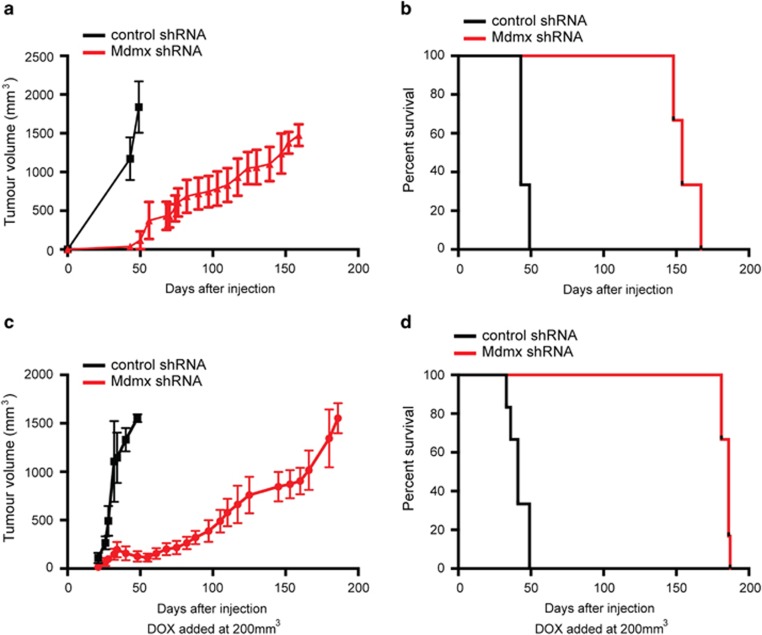
KD of Mdmx attenuated the development and progression of MCF-7-derived xenograft tumors in mice. MCF-7 cells expressing Mdmx shRNA or control shRNA (vector alone) were injected into two mammary fat pads of each female mouse (*n*=6 per group). For the assay assessing prevention of tumor development, mice were pretreated 3 days before cell injection then again on the day of injection with DOX (0.4 mg/ml) intraperitoneally, followed by DOX in drinking water (2 mg/ml). Tumor volume was measured every 3 days following the first appearance (**a**) and mice were culled and analyzed when tumor volume reached ethical size (**b**). The median survival for the control group was 43 days, whereas survival for the group expressing Mdmx shRNA was 154 days (Mantel–Cox test, *P*=0.0006). For the treatment assay, the DOX treatment started when tumor size reached 200 mm^3^. Tumor volume (**c**) and survival of mice are plotted (**d**). The median survival for the control group was 41 days, whereas that of Mdmx shRNA-expressing group was 186 days (Mantel–Cox test, *P*=0.0008)

To determine whether the effects of Mdmx KD on cell growth and reactivation of p53 that were observed in cultured cells *in vitro* also occur *in vivo*, we employed the orthotopic xenograft transplantation approach in NOD/SCID/common−*γ*chain knockout (NSG) mice. Initially, we examined the effect of Mdmx KD on the establishment of the tumors. For this purpose, MCF-7 cells expressing either the conditional Mdmx KD or control lentiviral vector were orthotopically injected into contralateral mammary fat pads (numbers 4 and 9) of six NSG females and mice were treated with DOX through an IP injection followed by DOX-supplemented drinking water. Tumors in the control mice grew rapidly, with the sum of the two tumors per mouse reaching ethical size of 1500 mm^3^ by ~50 days post injection (Figure 4a) and were duly terminated (Figure 4b). Remarkably, by this time point none of the Mdmx KD mice developed tumors to any significant size (Figure 4a) and survived past 150 days (Figure 4b). These results demonstrate that downregulation of Mdmx dramatically extends survival of mice orthotopically transplanted with luminal BrCa cells. Therefore, Mdmx promotes establishment of wt p53-harboring BrCa tumors in this orthotopic xenograft model.

To mimic a more clinical setting, we examined the effect of Mdmx KD on established tumors. For this purpose, the same orthotopic xenograft model described above was employed, with the exception that DOX treatment was started when tumors reached 200 mm^3^. The DOX treatment had no effect on the volume of the control tumors, which grew to the ethical end point size by ~50 days post injection. In stark contrast, DOX treatment of Mdmx KD tumors resulted initially in tumor regression, followed by extreme growth retardation compared with the controls. Importantly, the Mdmx KD tumors reached their ethical end point at ~180 days post injection (Figure 4c), 130 days after control mice termination (Figure 4d). This dramatic result clearly demonstrates that downregulation of Mdmx efficiently impedes the growth of an established tumor of transplanted MCF-7 cells. This provides the first demonstration that, at least in this model, downregulation of Mdmx can be used to treat established BrCa luminal tumors.

### Downregulation of Mdmx induces cellular senescence *in vivo*

**Figure 5:**
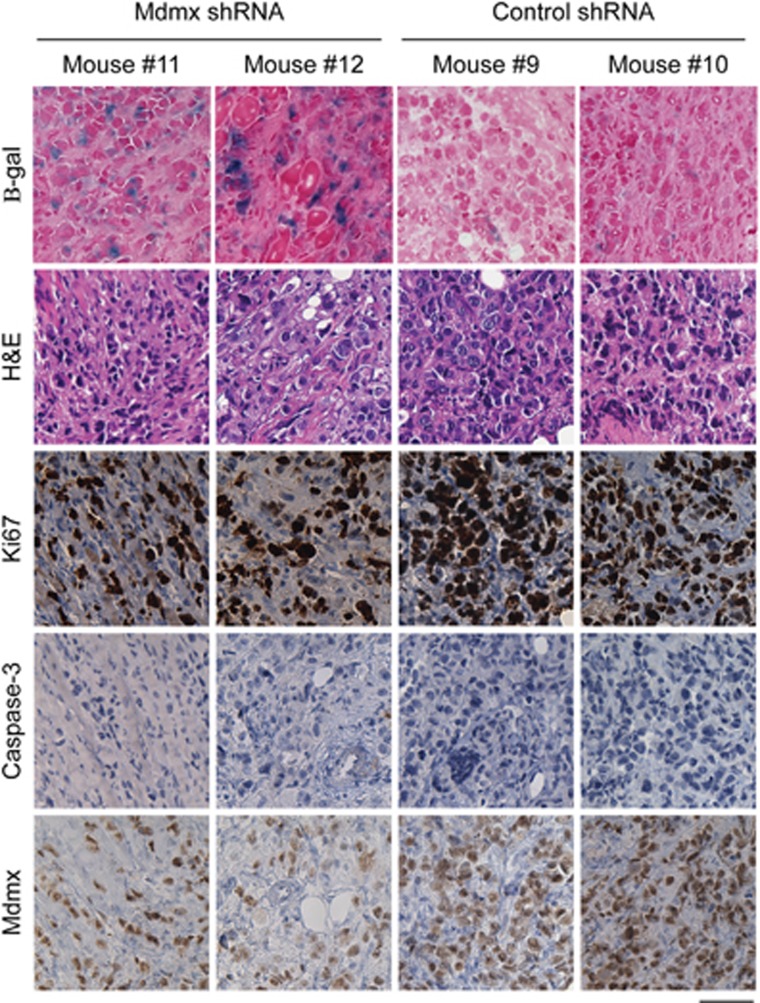
KD of Mdmx promoted cellular senescence in tumor xenografts *in vivo*. MCF-7 cells expressing Mdmx shRNA or control shRNA (shMdmx wobble control) were injected orthotopically into the mammary fat pads of NSG females (*n*=6) as described in Figure 4c treatment assay. Following 7 days of DOX treatment, mice were killed and tumors were either cryopreserved in preparation for *β*-gal staining (senescence marker) or formalin fixed and paraffin embedded for staining with hematoxylin and eosin (H&E; for morphological characterization) or IHC staining for Ki67 (proliferation marker), activated caspase-3 (apoptosis marker) and Mdmx, respectively. Mdmx KD was found to correspond with increased senescence and reduced ki67, even after only 7 days of treatment. The scale bar is 50 *μ*m. Representative tumors from two mice of each group mice are shown

To define the cellular mechanism by which Mdmx KD impedes the growth of MCF-7 cells *in vivo*, the treatment experiment described in Figures 4c and d was repeated with the exception that mice were subjected to DOX treatment for only 7 days before tumor tissues were collected for analyses. This time point was chosen, as it defined the narrow time window when the tumor size in both cohorts was sufficiently small for DOX infiltration, but large enough for accurate measurement. To identify the nature of the cellular response to Mdmx KD, samples were subjected to analysis of Ki67 as a proliferation marker, activated caspase-3 as a marker for apoptosis and *β*-gal for identification of cellular senescence. A distinct difference in the cellular response between control mice and the Mdmx KD cohort was evident after 7 days of DOX treatment. Notably, the Mdmx KD tumors revealed a significantly stronger staining for *β*-gal than the control group, implicating cellular senescence as the major cellular response to Mdmx KD (Figure 5). This notion is also supported by elevation in *p21* expression (as seen *in vitro* in Figures 2c and d), an important marker for cellular senescence.^21^ Consistently, reduction in Ki67 staining in Mdmx KD tumors was more evident than in the control group. Low levels of apoptosis, as defined by active caspase-3 staining, was measured in all samples, with no significant difference between the groups. This is consistent with senescence (as indicated by *β*-gal staining) as the major outcome in response to Mdmx KD. The morphology of the transplanted tumors was visualized by hematoxylin and eosin staining and, as expected, it was very similar between groups at this early time point. Reduction in Mdmx levels was identified as reduced staining intensity (not cellular proportion) in response to KD after 7 days, indicating that DOX was penetrating the tumors. We therefore conclude that Mdmx KD in MCF-7 cells induces cellular senescence *in vivo*.

### Growth inhibition through Mdmx KD is corroborated in wt p53 luminal MPE600 cell line

**Figure 6:**
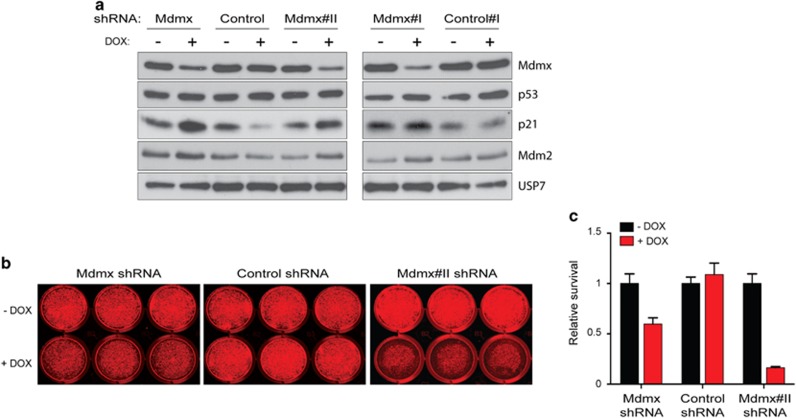
KD of Mdmx induced growth inhibition also in wt p53 luminal BrCa cell line MPE600. MPE600 cells transduced with either shMdmx constructs or shRNA controls were either not treated or treated with DOX (2.5 ng/ml) for 8 days and analyzed by western blotting (**a**) with protein staining for Mdmx, p53, p21, Mdm2 and USP7. Cell growth inhibition under the same conditions was demonstrated by a colony assay, representative plates are shown in **b** and the results are summarized in **c**

Reiteration of the growth inhibitory potency of Mdmx KD in an additional luminal BrCa cell line was considered important to establish its potential across this BrCa subtype. The wt p53 cell line MPE600 was subjected to Mdmx KD for 8 days (western blotting, Figure 6a). Mdmx KD was coincident with elevation of p21 and Mdm2 proteins levels, consistent with p53 transcriptional activity, where USP7 was used as a housekeeping control. Cell growth inhibition in response to 2.5 ng/ml DOX for 8 days was demonstrated in a colony assay (Figure 6b) and was identified to be statistically significant (Figure 6c).

### Basal-like cell line SKBR7 expressing wt p53 was also growth inhibited by Mdmx KD

**Figure 7:**
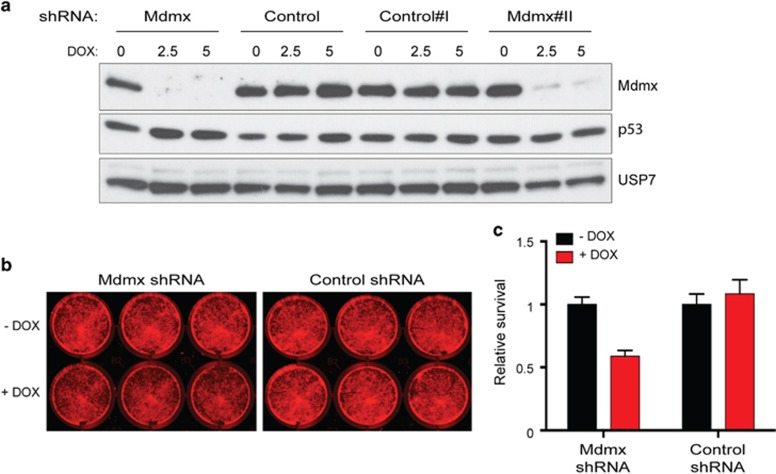
KD of Mdmx growth inhibited wt p53 basal-like SKBR7. Wt p53 basal-like SKBR7 cells transduced with either shMdmx constructs or shRNA controls were either not treated or treated with DOX (2.5 and 5 ng/ml) for 8 days and analyzed by western blotting (**a**) with protein detection of Mdmx, p53 and USP7. A colony assay of 2.5 ng/ml DOX-treated samples and controls demonstrated significant growth inhibition with Mdmx KD as visualized in **b** and as quantified in **c**

To identify whether high levels of Mdmx in additional BrCa subtypes could account for wt p53 tolerance, the basal-like BrCa cell line SKBR7 was subjected to Mdmx KD (2.5 ng/ml DOX, 8 days; western blotting, Figure 7a), causing profound growth inhibition (Figure 7b), in a statistically significant manner (Figure 7c). Together, these findings imply that Mdmx KD has a capacity to inhibit the growth of BrCas expressing wt p53, independent of BrCa subtype.

## Discussion

The molecular explanation for the relatively low p53 mutational frequency in luminal BrCas and increased incidence in basal-like subtypes in contrast^16, 18^ has only been partially described. The major molecular mechanisms underlying wt p53 status in cancer cells are elevation in the Mdm proteins levels (reviewed in Wade *et al.*^6^ and Marine *et al.*^10^) or a loss of the key activator ARF (reviewed in Sherr^22^). Screening for alterations in the *Mdm* genes in luminal BrCa revealed rare amplification of the *Mdm2* gene^14, 23^ and no selection for *Mdm2* SNP309 in primary tumors.^24^

Similar to our findings, Mdm2 nuclear staining was relatively frequent in BrCa biopsies (>50%)^25^ and also in ~40% of benign BrCa cases.^26^ However, deregulated Mdm2 linked to estrogen in this BrCa context cannot account for low p53 levels (as reported for MCF7, where estrogen-induced elevated Mdm2 did not correlate with reduced p53).^27^

As for Mdmx, there is no evidence for its involvement in heritable BrCa,^28^ and in somatic BrCa reports of amplifications of the *Mdmx* gene vary^14, 15^ (apparently dependent on the designated threshold). Our results are consistent with low-frequency involvement of Mdm2 in luminal BrCa, but revealed high Mdmx protein levels in all the luminal BrCa cases (Figure 1). Our FISH analyses identified only one sample with amplification of the *Mdmx* gene (Supplementary Figure 1); hence, we conclude that elevation in Mdmx expression is not due to gene amplification. Danovi *et al.*^15^ showed that the majority of BrCa cases did not express high Mdmx RNA levels, which support the notion that much of the elevation in Mdmx expression occurs at the protein level.

Unexpectedly, we found that normal breast ductal epithelial cells express elevated levels of Mdmx and Mdm2, correlating with low p53 levels. In contrast, the myoepithelial cells, stroma and fatty tissue lack Mdmx staining. Importantly, the breast ductal epithelial cells are the origin of malignant growth. Our study therefore demonstrates that during the transition to luminal malignancy, the levels of Mdmx and Mdm2 increase from an already high expression. This is consistent with the lack of gene amplification in most BrCa samples that we analyzed. The finding of elevated Mdm proteins in normal breast was unexpected, as it stands in sharp contrast to melanoma^13^ and gastric^29^ cancers, for example, where normal skin and the stomach have near to no detectable Mdmx levels.

As p53 is activated by oncogenic stresses, we argue that the maintenance of high levels of Mdm protein expression in the breast is selected for during tumorigenesis, to suppress the growth inhibition by p53. A central question raised by these observation is why are such elevated levels of the Mdm proteins required and why exclusively in the epithelial cells? High Mdm levels in the normal ductal epithelial cells have not been duly considered to our knowledge in studies of BrCa to date (despite indication of staining in the normal breast in The Protein Altas^30^). These findings are likely to have important consequences for therapy as discussed below.

Mdmx has been targeted in MCF-7 cells by RNAi^15, 20, 31^ and by small-molecule inhibitors,^32^ causing significantly reduced cell proliferation. Our *in vitro* results (Figure 2) are consistent with these findings and support the p53 dependence (Figure 3). Importantly, by reiterating growth inhibition induced by Mdmx KD in an additional wt p53 luminal BrCa line (Figure 6), our findings demonstrate the broader implications of this phenomena across this BrCa subtype.

We have extended the *in vitro* findings to a mouse model demonstrating that Mdmx KD efficiently attenuates the establishment of orthotopic transplantation of MCF-7 cells (Figure 4), regresses established tumors and attenuates tumor regrowth, which together substantially prolongs life by almost 19 weeks (Figure 4). Validating the effect of Mdmx KD in patient-derived xenografts (PDXs) is desirable; however, the ‘take-rate' of luminal BrCA PDXs is very low,^33^ having an impact on the feasibilities of such experiments. The predominant response in these tumors is cellular senescence (Figure 5), which is consistent with elevated p21 expression following Mdmx KD (Figure 2). Previous studies targeting Mdmx in MCF-7 cells using small-molecule inhibitors resulted in the activation of p53-induced cell death^32, 34^ or cell cycle arrest.^31^ However, in both studies the assays were performed *in vitro*, whereas our analysis was performed on *in vivo*-derived tumors. Therefore, we provide the first demonstration of the role of cellular senescence in response to targeting Mdmx in BrCa *in vivo*.

Significantly, these studies for the first time extended beyond the luminal subtype to basal-like BrCa and demonstrated that in a wt p53 background, Mdmx KD caused potent growth inhibition (SKBR7; Figure 7). These findings are profound, as they demonstrate that the growth inhibitory capacity of Mdmx KD is not restricted by BrCa subtype and thus has potential therapeutic relevance to all wt p53 BrCas.

Approximately 50% of human cancers express wt p53. The proof-of-concept of restoration of wt p53 tumor-suppressive function has been demonstrated in a series of elegant papers, using multiple mouse models with conditional p53 expression.^35, 36, 37^ As Mdm2 and Mdmx are the key inhibitors of p53, cancers in which either or both Mdm proteins are deregulated opens an attractive opportunity to restore p53 activity by targeting either one or both inhibitors. Targeting Mdm2 as a means to restore p53 function has attracted the major efforts to date.^38^ However, clinical trials revealed significant side effects due to on-target effects of Mdm2 inhibition in driving hematological toxicity as well as toxicity in the digestive system (e.g., see Ray-Coquard *et al.*^39^). These issues lend strong support for targeting Mdmx as an alternative approach to restore p53 activities (reviewed in Marine^40^). The proof-of-concept for targeting Mdmx has been successfully demonstrated in multiple cultured cell culture models and, importantly, in an *in vivo* mouse model, which demonstrated tolerance of mouse tissues to Mdmx deletion.^6, 40, 41^ We have now extended it to an *in vitro* and *in vivo* experimental model for wt p53 BrCa cells. The important distinction between BrCa cell sensitivity to Mdmx KD (as seen in this study and in others^15, 31, 32, 34^) compared with the apparent tolerance of normal cells^41^ implies that BrCa and other cancer cells with elevated Mdmx are hypersensitized to p53 activation. This phenomenon has been demonstrated in a wide range of cancers, where p53 has been restored (see Martins *et al.*,^35^ Ventura *et al.*^36^ and Xue *et al.*^37^) and supports the implications of our study that targeting Mdmx is a valid approach for treating BrCas harboring wt p53.

Multiple approaches have been developed to protect p53 from the inhibitory effects of Mdmx (reviewed in Wade *et al.*^6^ and Khoo *et al.*^38^). Targeting the p53–Mdmx interaction predominates using the following: small molecules (SJ-172550, WK298 and RO-5963), stapled peptides (SAH-p53-8, sMTide-02/O2a and ATSP-7041) and other modified peptides (pDI, PMI and PMI-N8A). Alternative approaches to reduce Mdmx expression levels include small molecules such as NSC207895, which is related to DNA-damaging agents, or 17-AAG, an HSP-90 inhibitor that destabilizes Mdmx by unknown mechanisms (reviewed in Wade *et al.*^6^ and Khoo *et al.*^38^). Some of these small molecules and peptides have a dual specificity for Mdm2 and Mdmx, albeit often with different affinities. The potency of dual targeting in BrCa is suggested by an *in vitro* study,^42^ but awaits testing *in vivo* with precision therapeutic agents.

## Conclusions

Our study demonstrates that Mdmx is highly expressed in ductal epithelial breast cells, but not in other breast cells, and is further elevated in BrCa. Downregulation of Mdmx alone is sufficient to significantly attenuate the establishment of tumor growth and the progression of established tumors. Overall, our findings suggest that targeting Mdmx is an attractive strategy for the treatment of BrCas that express wt p53 (~70% of all cases).

## Materials and Methods

All chemicals and reagents used in this study were purchased from Sigma-Aldrich, NSW, Australia, unless otherwise indicated. Analytical grade solvents and double-distilled water were used for all experiments, which were performed in triplicate and undertaken at least three times.

### Cell culture

The BrCa cell line MCF-7 was acquired from ATCC (www.atcc.org) and maintained in Dulbecco's modified Eagle's medium (Gibco, Invitrogen, VIC, Mount Waverley, Australia) supplemented with 10% fetal calf serum (FCS) (Gibco) and 0.1% penicillin/streptomycin. BrCa cell lines SKBR7 and MPE600 were a gift from Mieke Schutte/John Martens (Erasmus MC, Rotterdam, The Netherlands) and were maintained in RPMI 1640 (Invitrogen) supplemented with 10% FCS and antibiotics. All cells were incubated at 5% CO_2_, 37 °C. All cells express wt p53 and elevated levels of Mdmx were previously demonstrated.^19^

### Inducible lentiviral shRNA constructs and viral production

A third-generation lentiviral vector, FH1t with GFP tag^43^ was harnessed to generate shRNA to KD Mdmx (shMdmx: forward (Fwd) strand: 5′-TCCC ACAGTCCTTCAGCTATTTCAT TTCAAGAGA ATGAAATAGCTGAAGGACTGT TTTTTC 3′ and reverse (Rev) strand: 5′-TCGAGAAAAA ACAGTCCTTCAGCTATTTCAT TCTCTTGAA ATGAAATAGCTGAAGGACTGT-3′) and for the control shRNA (shMdmxWobble: Fwd strand: 5′-TCCC ACCGTCCGCAAGCTATGTCAT TTCAAGAGA ATGACATAGCTTGCGGACGGT TTTTTC-3′ and Rev strand: 5′-TCGAGAAAAA ACCGTCCGCAAGCTATGTCAT TCTCTTGAA ATGACATAGCTTGCGGACGGT-3′). Confirmation was provided by additional Mdmx shRNA constructs (shMdmx#I: Fwd strand: 5′-TCCC GTGCAGAGGAAAGTTCCAC TTCAAGAGA GTGGAACTTTCCTCTGCAC TTTTTC-3′ and Rev strand: 5′-TCGAGAAAAA GTGCAGAGGAAAGTTCCAC TCTCTTGAA GTGGAACTTTCCTCTGCAC-3′ shMdmx#II: Fwd strand: 5′-TCCC AGTCAAGACCAACTGAAGC TTCAAGAGA GCTTCAGTTGGTCTTGACT TTTTTC-3′ and Rev strand: 5′-TCGAGAAAAA AGTCAAGACCAACTGAAGC TCTCTTGAA GCTTCAGTTGGTCTTGACT-3′ shMdmx#III: Fwd strand: 5′-TCCC GAATCTCTTGAAGCCATGT TTCAAGAGA ACATGGCTTCAAGAGATTC TTTTTC-3′ and Rev strand: 5′-TCGAGAAAAA GAATCTCTTGAAGCCATGT TCTCTTGAA ACATGGCTTCAAGAGATTC-3′). The additional shControl I was directed toward mouse Mdmx sequence and does not knock down human Mdmx Fwd strand: 5′-TCCC GAATCTTGTTACATCAGCT TTCAAGAGA AGCTGATGTAACAAGATTC TTTTTC-3′ and Rev strand: 5′-TCGAGAAAAA AGCTGATGTAACAAGATTC TCTCTTGAA ACATGGCTTCAAGAGATTC-3′. Viruses were generated and cells transduced essentially as described,^43^ with CaPO4 transfection replaced by PEI (as per the manufacturer's instructions).

### Western blot analysis

Western blot analysis was undertaken as previously described.^44^ Immunoblotting was performed with antibodies to human as follows: p53 (DO-1; 1801; a kind gift of Sir D. Lane), Mdmx (mouse clone 8C6; Millipore, Billerica, MA, USA; BL1285 Bethyl Laboratories, Inc., Montgomery, TX, USA), Mdm2 (SMP14; Santa Cruz Biotechnology, Inc., Dallas, TX, USA; 3G9 and Millipore), HSP 60 (rabbit; sc-13966; Santa Cruz Biotechnology, Inc.), USP7 (BL1285 Bethyl Laboratories) and p21 (sc-397; Santa Cruz Biotechnology, Inc.; CP64 Millipore).

### Flow cytometry analyses

Cells were cultured in doxycyclin (DOX) for induction of the shRNAs and were collected for live cell counts essentially as described previously,^45^ with the exception that To-pro-3-iodide (Life Technologies, Thermo Scientific, VIC, Mulgrave, Australia) was employed for DNA staining (according to the manufacturer's instructions) and volumetric cell enumeration was undertaken on the FACSVERSE (BD Biosciences, North Ryde, NSW, Australia). Cell cycle analyses were undertaken using the same facilities following the addition of 0.1% Triton X-100, to permeabilize the plasma membranes.

### Quantitative real-time RT-PCR

Total RNA was extracted using TRIzol (Life Technologies, Thermo Scientific) according to the manufacturer's protocol. mRNA was reverse transcribed using M-mlv Reverse transcriptase (Promega, Alexandria, NSW, Australia) and QPCR was performed on the cDNA using SYBR green-reagent (Applied Biosystems, Scoresby, VIC, Australia) and designated primer sets.

Hu UBC Fwd: 5′-ATTTGGGTCGCGGTTCTTG-3′

Hu UBC Rev: 5′-TGCCTTGACATTCTCGATGGT-3′

Hu p21 Fwd: 5′-GAGGCCGGGATGAGTTGGGAGGAG-3′

Hu p21 Rev: 5′-CAGCCGGCGTTTGGAGTGGTAGAA-3′

Hu Mdmx Fwd: 5′-CAGCAGGTGCGCAAGGTGAA-3′

Hu Mdmx Rev: 5′-CTGTGCGAGAGCGAGAGTCTG-3′

The expression of each gene was calculated based on the cycle threshold (CT), set within the linear range of DNA amplification. The expression (arbitrary units) was calculated as the relative transcript abundance (RTA) by: RTA=10 000/(2ΔCT), where ΔCT=CT (gene of interest)−CT(UBC).

### Colony assays

For colony assay, 10 000 (SKBR7) or 20 000 (MPE600) cells were seeded into 12-well plates and allowed to recover overnight before the addition of DOX (2.5 ng/ml). Nine days later, cells were fixed with 4% paraformaldehyde and stained with crystal violet (0.05% aqueous; 30 min). Plates were rinsed, dried and scanned on the Licor Odyssey (Mulgrave, VIC, Australia). Intensity of the signal was determined with the Image Studio software (Mulgrave, VIC, Australia).

### Orthotopic tumor studies

Female NOD SCID *γ*-IL2R *γ-*chain (NSG) mice at ~6–8 weeks were orthotopically injected into contralateral mammary fat pads (numbers 4 and 9). The gene KD was affected by DOX administration initially through IP injection (40 *μ*g/100 *μ*l, with simultaneous DOX supplementation in the drinking water (2 mg/ml). Mice were aged during which time their tumors were measured, and at their ethical endpoints they were killed per institutional guidelines. Necropsies were performed, and tumor and control tissues were collected and divided for protein and RNA analysis and microscopy. All procedures were conducted in accordance with the Institute for Laboratory Animal Research Guide: The Care and Use of Laboratory Animals. All animal work was performed with approval from the Peter MacCallum Cancer Centre Animal Experimentation Ethics Committee.

### Microscopy analyses

In preparation for examination of fixed tissues, tumors were preserved in 10% neutral buffered formalin. Fixed tissues were processed for paraffin embedding, sectioned and stained. Immunohistochemical staining was performed with antibodies to human p53 (Leica Biosystems, Newcastle Upon Tyne, UK; Novocastra mouse monoclonal DO-7), Mdmx (Bethyl Laboratories, Inc.; A300 287A), Mdm2 (Thermo Scientific, Neo Markers, Fremont, CA, USA), caspase-3 (R&D Systems, Minneapolis, MN, USA; Monoclonal Mouse 84803) and ki67 (Cell Marque, Sigma-Aldrich company, Carlsbad, CA, USA; rabbit monoclonal, SP6). Histopathological analyses were performed by an onco-pathologist (JMP). Photographs were taken using a using a BX-50 microscope with Leica DFC290HD camera (Olympus, Notting Hill, VIC, Australia) and Leica Application Suite V3.8.0 software. IHC scoring was undertaken using BX-41 microscope (Olympus).

Fresh-frozen tissues were prepared from OCT-embedded tissues snap frozen in isopentane liquid nitrogen vapors and cryostat sections were stained for *β*-gal (essentially as in Dimri *et al.*^46^). Images were visualized using a BX- 51 microscope (Olympus). *β*-Gal staining of cultured cells was performed as published.^46^ Images were visualized using a Zeiss Axio Vert.A1 microscope (Zeiss, Peabody, MA, USA) and acquired using SPOT Version 4.7 software (Diagnostic Instruments).

### Tissue microarray

BrCa biopsies from archived patient samples from the Peter MacCallum Cancer Institute, Melbourne, were compiled into a BrCa TMA. Each tissue core was of 1-mm diameter^47^ and was classified into luminal, basal, HER2 and null subtypes according to immunohistochemical profiling.^48^ TMA were immunostained using the antibodies listed above. Samples were scored for both the proportion of cells stained and the intensity of staining. Staining intensity was evaluated on an escalating scale ranging from 0 to 3, whereas the proportion of cells stained was according to the following scale: 0 is negative; 1: <10% stained; 2: 10–50% stained; 3: 50–80% stained; and 4: at least 80%. A combined histoscore was calculated by adding the value of the intensity to the proportion of cells stained with a final maximum score of 8. The TMA consisted for 81 biopsies samples: 50 luminal (62%), 20 Her2 (25%), 11 basal (13%) and a panel of normal breast tissues; these were analyzed for the IHC staining.

### Fluorescence *in situ* hybridization

Analyses were undertaken essentially as published.^49^ Target detection used were as follows: Vysis LSI *MDM2* Spectrum Orange Probe (Abbott Molecular, Des Plaines, IL, USA) and Vysis CEP 12 (D12Z3) Spectrum Green Probe (Abbott Molecular) or Poseidon *MDM4* (1q32) and SE 1 Control Probe (Kreatech Diagnostics, Amsterdam, The Netherlands). Fifty tumor nuclei per case were assessed for each case using the following criteria: a case was considered amplified if the ratio was >2.0 and not amplified if the ratio was≤2.0. This study was carried out according to the provisions of the Helsinki Declaration of 1975 and was reviewed and approved by the Peter MacCallum Cancer Centre Ethics Committee. For FISH analysis, *Mdm2* gene amplification analyses 76 biopsy cores were analyzed: luminal *n*=36; Her2 *n*=12; and basal *n*=9. For *Mdmx* gene amplification, 80 biopsy cores were analyzed: luminal *n*=49; Her2 *n*=19; and basal *n*=12 (where numbers differ slightly from IHC due to core dropout during FISH staining).

## Abbreviations

*β*-gal: *β*-galactosidase
CT: cycle threshold
DOX: doxycycline
FISH: fluorescence *in situ* hybridization
Fwd: forward
KD: knockdown
NSG: NOD SCID *γ*-IL2R *γ-*chain
PFT*α*: pifithrin-*α*
Rev: reverse
RTA: relative transcript abundance
TMA: tissue micro-array
Wt: wild type
