# Supplementary figures and images for: Targeting Mdmx to treat breast cancers with wild-type p53
Source: Cell Death Dis. 2015 Jul 16;6(7):e1821–. doi: 10.1038/cddis.2015.173 (PMC4650725; doi:10.1038/cddis.2015.173)

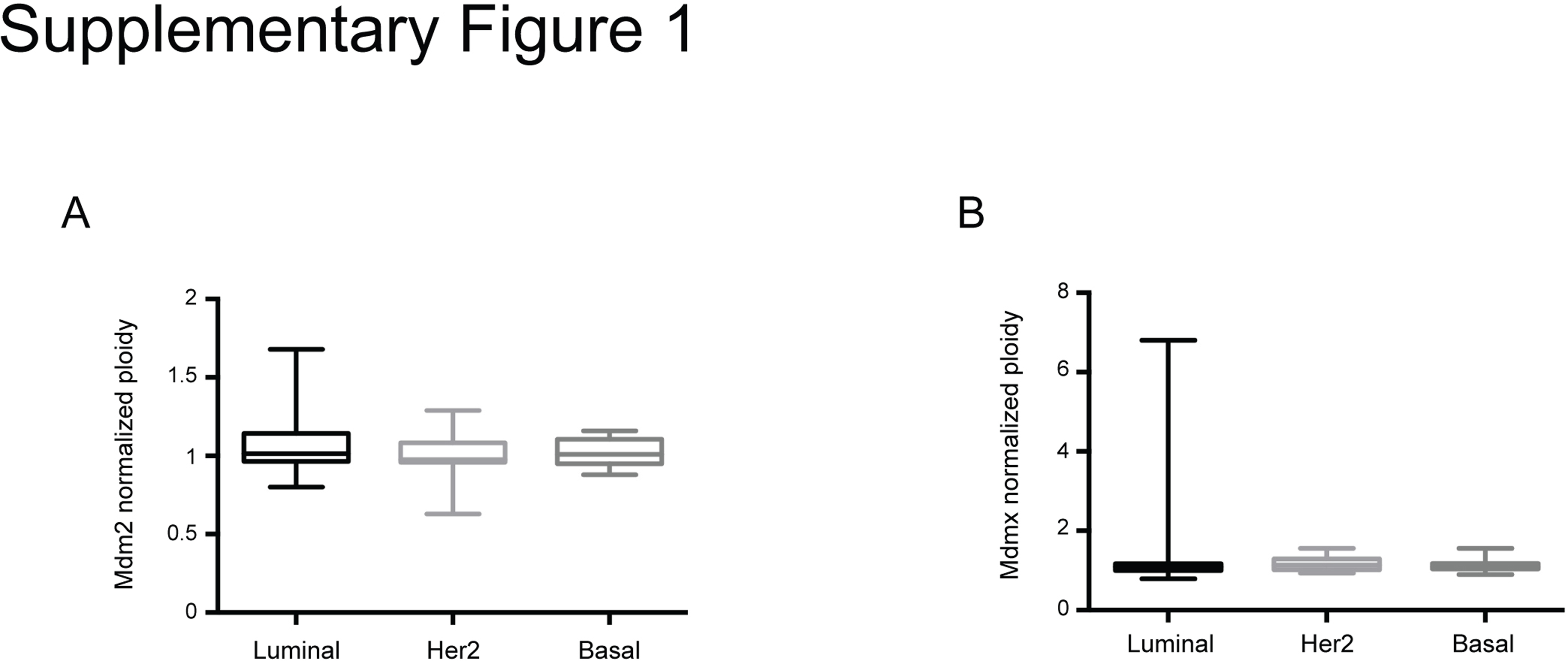

Supplement: Supplementary Figure 1 [file cddis2015173x1.tif]

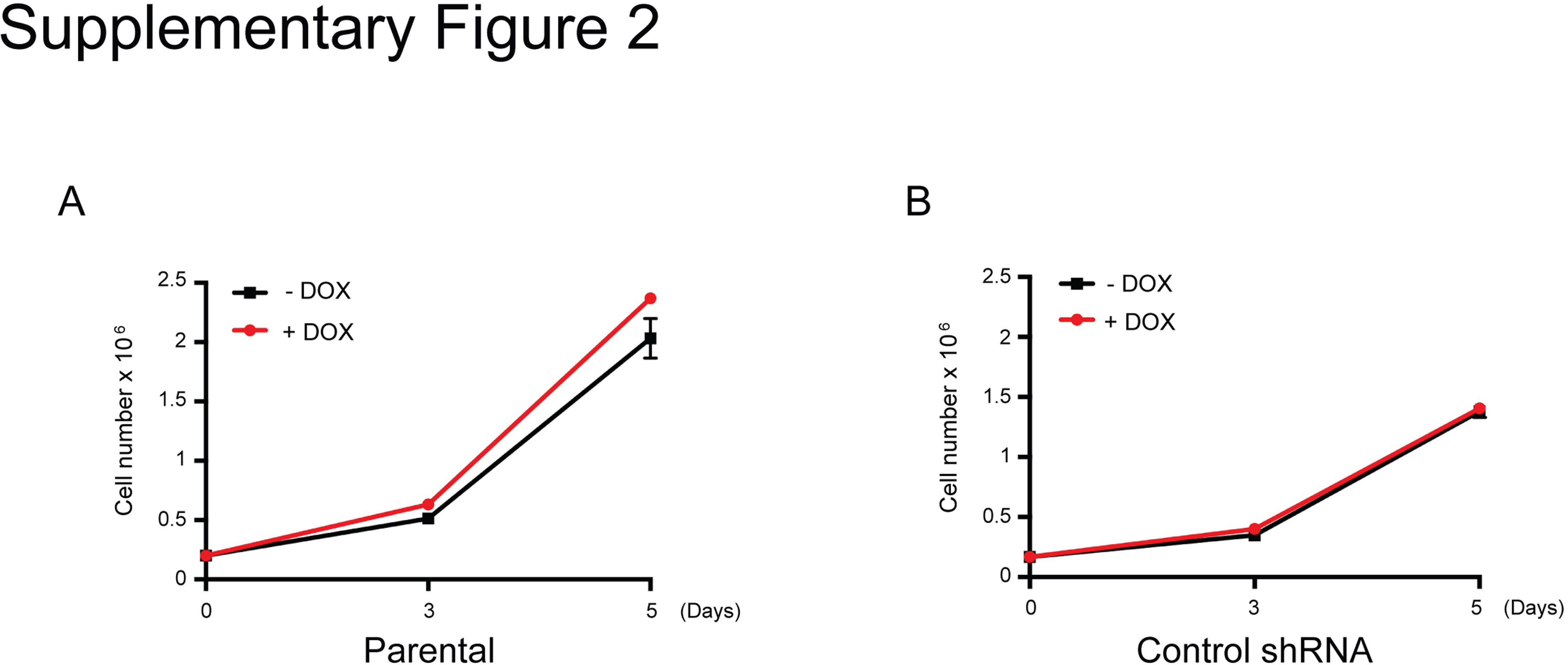

Supplement: Supplementary Figure 2 [file cddis2015173x2.tif]

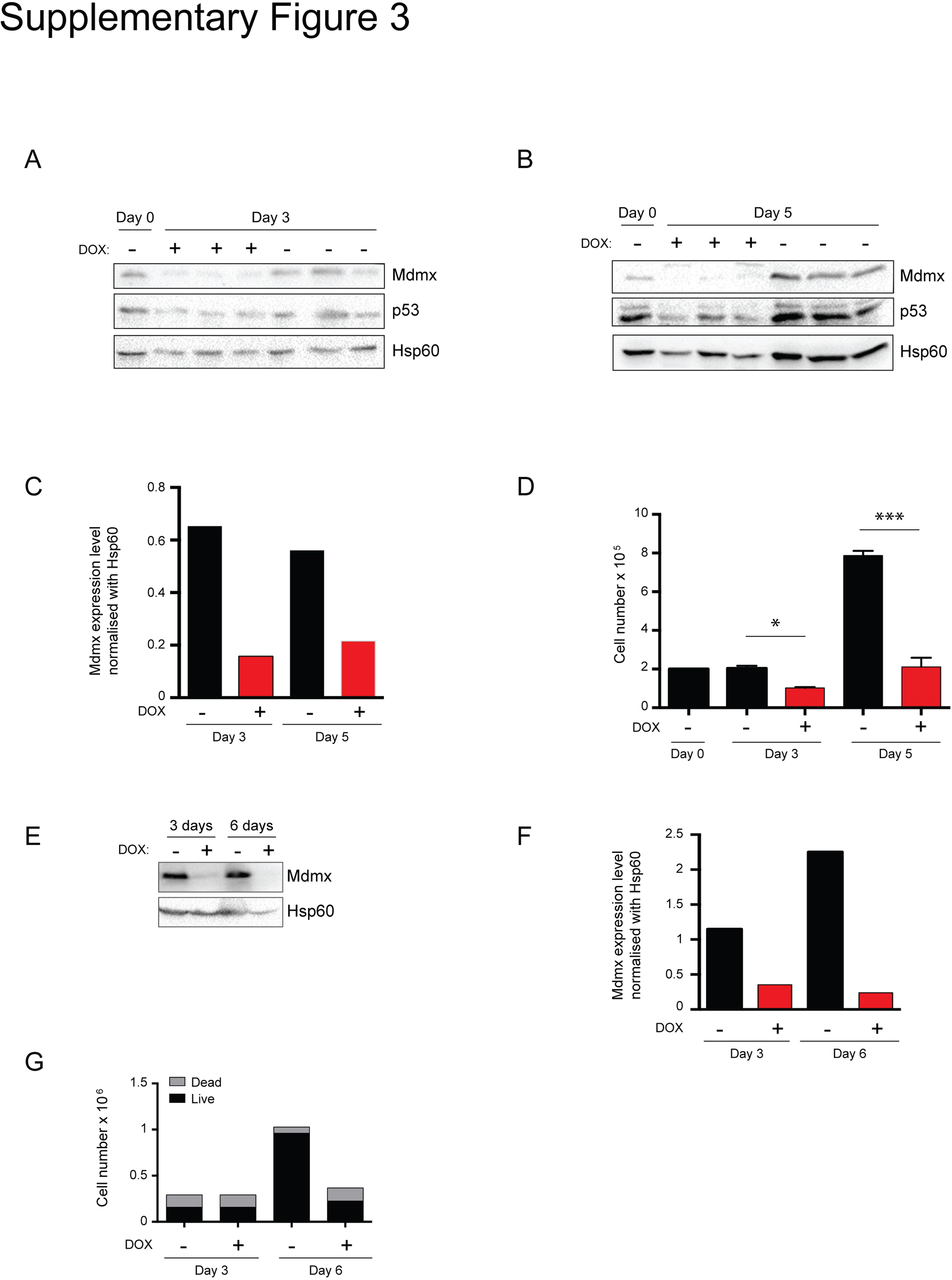

Supplement: Supplementary Figure 3 [file cddis2015173x3.tif]
