# Supplementary material for: Targeting Mdmx to treat breast cancers with wild-type p53
Source: Cell Death Dis. 2015 Jul 16;6(7):e1821–. doi: 10.1038/cddis.2015.173 (PMC4650725; doi:10.1038/cddis.2015.173)
Supplement: Supplementary Figure Legends [file cddis2015173x4.doc]

**SUPPLEMENTARY FIGURE LEGENDS**

**Supplementary Figure 1.** FISH analysis identified Mdm2 and Mdmx gene amplification as a rare event in BrCa.

FISH analyses were undertaken on the BrCa TMA for Mdm2 (A) and Mdmx (B). Only a single sample exhibited significant elevation and this was a luminal BrCa sample with elevated Mdmx expression. Data is normalized to one, indicating normal ploidy (n=2).

**Supplementary Figure 2**. DOX treatment did not affect the growth of parental MCF-7 cells or cells transduced with a control shRNA.

MCF-7 cells were either untreated or treated with doxycycline (DOX; 80ng/ml), and cell numbers were counted at 3 and 5 days: MCF-7 cells parental, untransduced cells (A) and Control shRNA (empty vector control; B respectively). Neither cell line exhibited significant variation in response to DOX treatment.

**Supplementary Figure 3**. Downregulation of Mdmx in MCF-7 cells with distinct Mdmx shRNA sequence (referred to as #I and #II, respectively) also attenuated Mdmx levels and cell proliferation confirming the reproducibility of the effect with multiple Mdmx shRNA sequences.

Mdmx levels were reduced by Mdmx shRNA #I following DOX induction compared to their untreated counterparts, as shown for biological triplicates after 3 days of treatment (A) and 5 days (B) and quantified by densitometry (C). Live cell numbers for these samples clearly indicated growth inhibition to be a consequence of Mdmx KD in these cells (D). P values were calculated by the Student’s t test (P=0.0189 after 3 days Dox; P=0.0004 after 5 days DOX).

Additionally, Mdmx levels were reduced in MCF-7 cells transduced with Mdmx shRNA #II treated with DOX for 3 and 6 days respectively (E) and confirmed by densitometric quantification (F). The viability of the cells was assessed by Trypan dye exclusion (G).
